# Gaining Mathematical Understanding: The Effects of Creative Mathematical Reasoning and Cognitive Proficiency

**DOI:** 10.3389/fpsyg.2020.574366

**Published:** 2020-12-18

**Authors:** Bert Jonsson, Carina Granberg, Johan Lithner

**Affiliations:** ^1^Department of Applied Educational Science, Umeå University, Umeå, Sweden; ^2^Umeå Mathematics Education Research Center, Umeå, Sweden; ^3^Department of Science and Mathematics Education, Umeå University, Umeå, Sweden

**Keywords:** creative mathematical reasoning, cognitive proficiency, working memory, fluid intelligence, rote learning

## Abstract

In the field of mathematics education, one of the main questions remaining under debate is whether students’ development of mathematical reasoning and problem-solving is aided more by solving tasks with given instructions or by solving them without instructions. It has been argued, that providing little or no instruction for a mathematical task generates a mathematical struggle, which can facilitate learning. This view in contrast, tasks in which routine procedures can be applied can lead to mechanical repetition with little or no conceptual understanding. This study contrasts Creative Mathematical Reasoning (CMR), in which students must construct the mathematical method, with Algorithmic Reasoning (AR), in which predetermined methods and procedures on how to solve the task are given. Moreover, measures of fluid intelligence and working memory capacity are included in the analyses alongside the students’ math tracks. The results show that practicing with CMR tasks was superior to practicing with AR tasks in terms of students’ performance on *practiced test tasks* and *transfer test tasks*. Cognitive proficiency was shown to have an effect on students’ learning for both CMR and AR learning conditions. However, math tracks (advanced versus a more basic level) showed no significant effect. It is argued that going beyond step-by-step textbook solutions is essential and that students need to be presented with mathematical activities involving a struggle. In the CMR approach, students must focus on the relevant information in order to solve the task, and the characteristics of CMR tasks can guide students to the structural features that are critical for aiding comprehension.

## Introduction

Supporting students’ mathematical reasoning and problem-solving has been pointed out as important by the National Council of Teachers of Mathematics (NCTM; 26T^1^). This philosophy is reflected in the wide range of mathematics education research focusing on the impact different teaching designs might have on students’ reasoning, problem-solving ability, and conceptual understanding (e.g., Coles and Brown, 2016; Lithner, 2017). One of the recurrent questions in this field is whether students learn more by solving tasks with given instructions or without them: “The contrast between the two positions is best understood as a continuum, and both ends appear to have their own strengths and weaknesses” (Lee and Anderson, 2013, p. 446).

It has been argued that providing students with instructions for solving tasks lowers the cognitive demand and frees up resources that students can use to develop a conceptual understanding (e.g., worked example design; Sweller et al., 2011). In contrast, other approaches argue that students should not be given instructions for solving tasks; one example is Kapur (2008, 2010) suggestion of “ill-structured” task design. With respect to the latter approach, Hiebert and Grouws (2007) and Niss (2007) emphasize that providing students with little or no instruction generates a struggle (in a positive sense) with important mathematics, which in turn facilitates learning. According to Hiebert (2003) and Lithner (2008, 2017), one of the most challenging aspects of mathematical education is that the teaching models used in schools are commonly based on mechanical repetition, following step-by-step methods, and using predefined algorithms—methods that are commonly viewed as rote learning. Rote learning (i.e., learning facts and procedures) can be positive, as it can reduce the load on the working memory and free up cognitive resources, which can be used for more cognitively demanding activities (Wirebring et al., 2015). A typical example of rote learning is knowledge of the multiplication table, which involves the ability to immediately retrieve “7 × 9 = 63” from the long-term memory; this is much less cognitively demanding than calculating 7 + 7 + 7 + 7 + 7 + 7 + 7 + 7 + 7. However, if teaching and/or learning strategies are solely based on rote learning, students will be prevented from developing their ability to struggle with important mathematics, forming an interest in such struggles, gaining conceptual understanding, and finding their own solution methods.

Indeed, several studies have shown that students are mainly given tasks that promote the use of predetermined algorithms, procedures, and/or examples of how to solve the task rather than opportunities to engage in a problem-solving struggle without instruction (Stacey and Vincent, 2009; Denisse et al., 2012; Boesen et al., 2014; Jäder et al., 2019). For example, Jäder et al. (2019) examined mathematics textbooks from 12 countries and found that 79% of the textbook tasks could be solved by merely following provided procedures, 13% could be solved by minor adjustments of the procedure, and only 9% required students to create (parts of) their own methods (for similar findings, also see Pointon and Sangwin, 2003; Bergqvist, 2007; Mac an Bhaird et al., 2017). In response to these findings, Lithner (2008, 2017) developed a framework arguing that the use of instructions in terms of predefined algorithms has negative long-term consequences for the development of students’ conceptual understanding. To develop their conceptual understanding, students must instead engage in creating (parts of) the methods by themselves. This framework, which addresses algorithmic and creative reasoning, guides the present study.

### Research Framework: Algorithmic and Creative Mathematical Reasoning

In the Lithner (2008) framework, task design, students’ reasoning, and students’ learning opportunities are related. When students solve tasks using provided methods/algorithms, their reasoning is likely to become imitative (i.e., using the provided method/algorithm without any reflection). Lithner (2008) defines this kind of reasoning as Algorithmic Reasoning (AR), and argues that AR is likely to lead to rote learning. In contrast, when students solve tasks without a provided method or algorithm, they are “forced” to struggle, and their reasoning needs to be—and will become—more creative. Lithner denotes this way of reasoning as Creative Mathematical Reasoning (CMR) and suggests that CMR is beneficial for the development of conceptual understanding. It is important to note that creativity in this context is neither “genius” nor “exceptional novelty;” rather, creativity is defined as “the creation of mathematical task solutions that are original to the individual who creates them, though the solutions can be modest” (Jonsson et al., 2014, p. 22; see also Silver, 1997; Lithner, 2008; for similar reasoning). Lithner (2008) argues that the reasoning inherent in CMR must fulfill three criteria: (i) *creativity*, as the learner creates a previously unexperienced reasoning sequence or recreates a forgotten one; (ii) *plausibility*, as there are predictive arguments supporting strategy choice and verification arguments explaining why the strategy implementation and conclusions are true or plausible; and (iii) *anchoring*, as the learner’s arguments are anchored in the intrinsic mathematical properties of the reasoning components.

Previous studies have shown that students practicing with CMR outperform students practicing with AR on test tasks (Jonsson et al., 2014; Jonsson et al., 2016; Norqvist, 2017; Norqvist et al., 2019). Jonsson et al. (2016) investigated whether the effects of effortful struggle or overlapping processes based on task similarity (denoted as transfer appropriate processing, or TAP; Franks et al., 2000) underlie the effects of using CMR and AR. The results did reveal effects of TAP for both CMR and AR tasks, with an average effect size (Cohens *d*; Cohen, 1992) of *d* = 0.27. While for effortful struggle, which characterizes CMR, the average effect size was *d* = 1.34. It was concluded that effortful struggle is a more likely explanation for the positive effects of using CMR than TAP.

In sum, the use of instructions in terms of predefined algorithms (AR) is argued to have negative long-term consequences on students’ development of conceptual understanding and to deteriorate students’ interest in struggling with important mathematics (e.g., Jäder et al., 2019). In contrast, the CMR approach requires students to engage in a effortful and productive struggle when performing CMR (e.g., Lithner, 2017). However, since the students that participated in previous studies were only given *practiced test tasks* (albeit with different numbers), the results may “merely” reflect memory consolidation without a corresponding conceptual understanding. If, after practice, students can apply their acquired reasoning to tasks not previously practiced, this would indicate a conceptual understanding.

In the present study, we investigate the effects of using AR and CMR tasks during practice, on subsequent test tasks, including both *practiced test tasks* and *transfer test tasks*. We are familiar with the large amount of transfer research in the literature and are aware that a distinction has been made between near transfer and far transfer tasks (e.g., Barnett and Ceci, 2002; Butler et al., 2017). In the present study, no attempt to distinguish between transfer and near transfer is made, we define transfer tasks as tasks that require a new reasoning sequence in order to be solved (see Mac an Bhaird et al., 2017 for a similar argument). These tasks are further described in the Methods section in conjunction with examples of tasks.

#### Mathematics and Individual Differences in Cognition

Domain-general abilities, such as general intelligence, influence learning across many academic domains, with mathematics being no exception (Carroll, 1993). General intelligence, which is commonly denoted as the ability to think logically and systematically, was explored in a prospective study of 70,000 students. Overall, it was found that general intelligence could explain 58.6% of the variation in performance on national tests at 16 years of age (Deary et al., 2007). Others have found slightly lower correlations. In a survey by Mackintosh and Mackintosh (2011), the correlations between intelligence quotient (IQ) scores and school grades were between 0.4 and 0.7. Fluid intelligence is both part of and closely related to general intelligence (Primi et al., 2010), and is recognized as a causal factor in an individual’s response when encountering new situations (Watkins et al., 2007; Valentin Kvist and Gustafsson, 2008) and solving mathematical tasks (Floyd et al., 2003; Taub et al., 2008). Moreover, there is a high degree of similarity between the mathematics problems used in schools and those commonly administered during intelligence tests that measure fluid cognitive skills (Blair et al., 2005).

Solving arithmetic task places demands on our working memory because of the multiple steps that often characterize math. When doing math, we use our working memory to retrieve the information needed to solve the math task, keep relevant information about the problem salient, and inhibit irrelevant information. Baddeley (2000, 2010) multicomponent working memory model is a common model used to describe the working memory. This model consists of the phonological loop and the visuospatial sketchpad, which, respectively, handle visuospatial and phonological information. These two sub-systems are controlled by the central executive and its executive components, updating, shifting, and inhibition (Miyake et al., 2000). In his model, Baddeley (2000) added the episodic buffer, which is alleged to be responsible for the temporary storage of information from the two sub-systems and the long-term memory. Individual differences in the performance of complex working memory tasks, which are commonly defined as measures of the working memory capacity (WMC), arise from differences in an individual’s cognitive ability to actively store, actively process, and selectively consider the information required to produce an output in a setting with potentially interfering distractions (Shah and Miyake, 1996; Wiklund-Hörnqvist et al., 2016).

There is a wealth of evidence and a general consensus in the field that working memory directly influences math performance (Passolunghi et al., 2008; De Smedt et al., 2009; Raghubar et al., 2010; Passolunghi and Costa, 2019). In addition, many studies have shown that children with low WMC have more difficulty doing math (Adam and Hitch, 1997; McLean and Hitch, 1999; Andersson and Lyxell, 2007; Szücs et al., 2014). Moreover, children with low WMC are overrepresented among students with various other problems, including problems with reading and writing (Adam and Hitch, 1997; Gathercole et al., 2003; Alloway, 2009). Raghubar et al. (2010) concluded that “Research on working memory and math across experimental, disability, and cross-sectional and longitudinal developmental studies reveal that working memory is indeed related to mathematical performance in adults and in typically developing children and in children with difficulties in math” (p. 119; for similar reasoning, also see Geary et al., 2017).

#### Math Tracks

A *math track* is a specific series of courses students follow in their mathematics studies. Examples might include a basic or low-level math track in comparison with an advanced math track. In Sweden, there are five levels of math, each of which is subdivided into parts a--c, ranging from basic (a) to advanced (c). That is, course 1c is more advanced than course 1b, and course 1b is more advanced than course 1a. In comparison with social science students, natural science students study math on a higher level and move through the curriculum at a faster pace. At the end of year one, natural science students have gone through courses 1c and 2c, while social science students have gone through course 1b. Moreover, natural science students that are starting upper secondary school typically have higher grades from lower secondary school than social science students^2^. Therefore, in the present study, it is reasonable to assume that natural science students as a group have better, more advanced mathematical pre-knowledge than social science students.

In the present study, we acknowledge the importance of both fluid intelligence and working memory and thus include a complex working task and a general fluid intelligence task as measures of cognitive proficiency. Furthermore, based on their curriculum, the students in this study were divided according to their mathematical tracks (basic and advanced), with the aim of capturing differences in mathematical skills.

This study’s hypotheses were guided by previous theoretical arguments (Lithner, 2008, 2017) and empirical findings (Jonsson et al., 2014, 2016; Norqvist et al., 2019). On this basis, we hypothesized that:

1. Practicing with CMR tasks would to a greater extent facilitate performance on *practiced tests tasks* than practicing with AR tasks.
2. Practice with CMR tasks would to a greater extent facilitate performance on *transfer test tasks* than practice with AR tasks.
3. Students that are more cognitively proficient would outperform those who are less cognitively proficient on both *practiced test tasks* and *transfer test tasks*
4. Students enrolled in advanced math tracks are likely to outperform those enrolled in basic math tracks on both *practiced test tasks* and *transfer test tasks*.

### Rationales for the Experiments

The three separate experiments presented below were conducted over a period of 2 years and encompassed 270 students. The overall aim was to contrast CMR with AR with respect to mathematical understanding. An additional aim was to contrast more cognitively proficient students with less cognitively proficient students and investigate potential interactions. The experiments progressed as a function of the experimental finding obtained in each experiment and were as such, not fully planned ahead. Experiment 1 was designed to replicate a previous study on *practiced test tasks* (Jonsson et al., 2014), and also introduced *transfer test tasks* with the aim of better capturing conceptual understanding. However, when running a between-subject design, as in experiment 1, there is a risk of non-equivalent group bias when compared with using a within-subject design. It was also hypothesized that the findings (CMR > AR) could be challenged if the students were provided with an easier response mode. It was therefore decided that experiment 2 should employ a within-subject design and use multiple-choice (MC) questions as the test format. After experiment 2, it was discussed whether the eight *transfer test tasks* used in experiment 2 were too few to build appropriate statistics and whether the MC test format did not fully capture students’ conceptual understanding because of the possibility of students using response elimination and/or guessing. Moreover, the total number of test tasks was 32 (24 *practiced test tasks* and eight *transfer test tasks*), and some students complained that there were too many test tasks, which may have affected their performance. It was therefore decided that experiment 3 should focus solely on *transfer test tasks*, thereby decreasing the total number of test tasks but increase the number of *transfer test tasks* without introducing fatigue. In experiment 3, we returned to short answers as a test format, thus restricting the possibility of students using response elimination and/or guessing.

## Materials and Methods

### Practice Tasks

**FIGURE 1:**
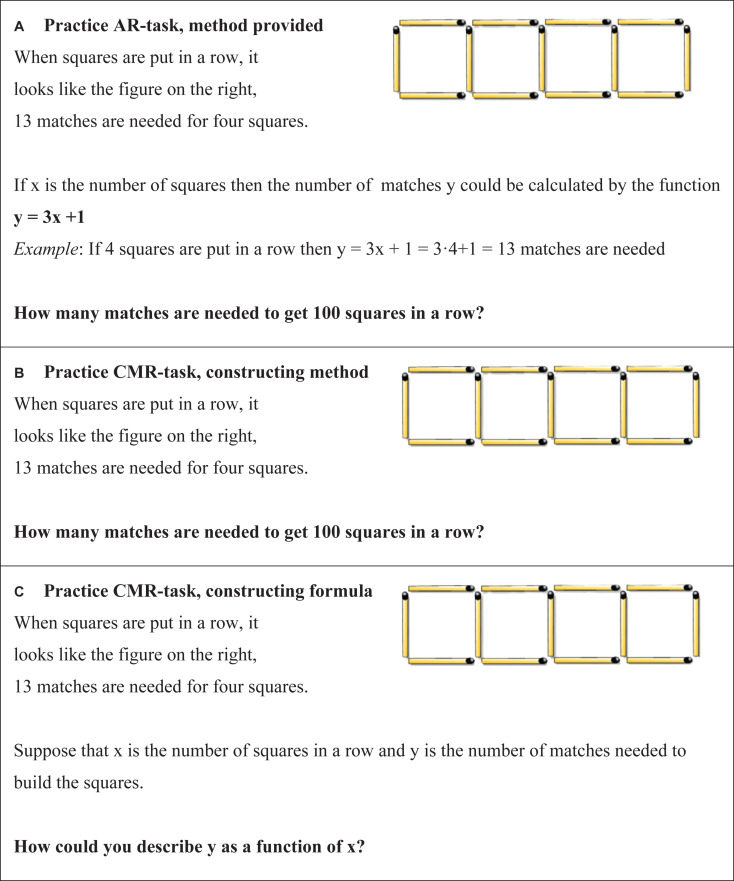
**(A–C)** Examples of AR and CMR *practice tasks* and how they were presented to the students on their laptop screen. **(A)** AR *practice task*; **(B)** CMR *practice task*; **(C)** CMR task asking for the formula.

A set of 35 tasks were pilot tested by 50 upper secondary school students. The aim was to establish a set of novel and challenging tasks that were not so complex that the students would have difficulty understanding what was requested. Twenty-eight of the 35 tasks fulfilled the criteria and were selected for the interventions. Each of the 28 tasks was then written as an AR task and as a CMR task, respectively (Figures 1A,B). The AR tasks were designed to resemble the design of everyday mathematical textbook tasks. Hence, each AR task provided the student with a method (a formula) for solving the task, an example of how to apply the formula, and a numerical test question (Figure 1A). The CMR tasks did not include any formulas, examples, or explanations, and the students were only asked to solve the numerical test questions (Figure 1B). Each of the 2 × 28 task sets (AR and CMR) included 10 subtasks, which only differed with respect to the numerical value used for the calculation. Although the number of task sets differed between the three experiments, there were 10 subtasks in each task set in all three experiments. Moreover, in each CMR task set, the third subtask asked students to construct a formula (Figure 1C). If the students completed all 10 subtasks, the software randomly resampled new numerical tasks until the session ended. This resampling ensured that the CMR and AR practice conditions lasted for the same length of time in all three experiments.

### Test Tasks

Test tasks that were the same as the *practice tasks* (albeit with different numbers) are denoted as “*practiced test tasks*” while the tasks that were different from the practice tasks are denoted as “*transfer test tasks*.”

#### Practiced Test Tasks

**FIGURE 2:**
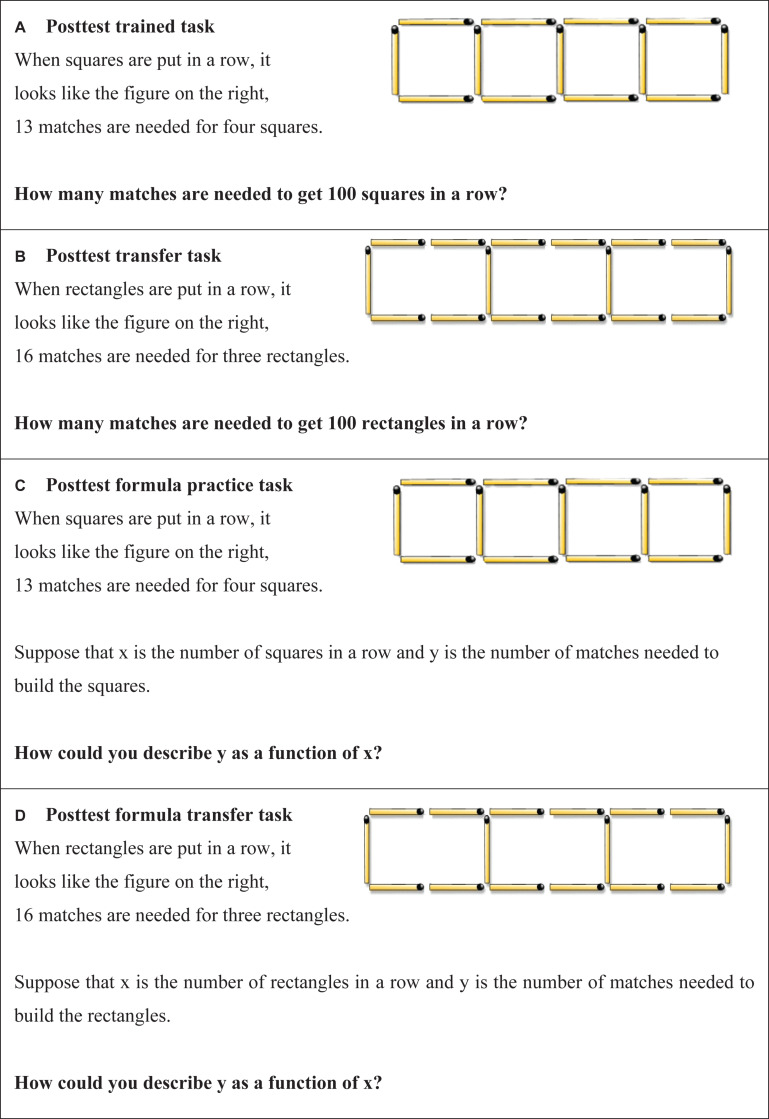
**(A–D)** Examples of test tasks and how they were presented to the students on their laptop screen. **(A,C)** *Practiced test tasks* and **(B,D)** *transfer test task*.

The layout of the *practiced test tasks* consisting of numerical- and formula tasks and can be seen in Figures 2A,C. The similarities between *practice tasks* and *practiced test tasks* may promote overlapping processing activities (Franks et al., 2000) or, according to the encoding specificity principle, provide contextual cues during practice that can aid later test performance (Tulving and Thomson, 1973). *Transfer test tasks* were therefore developed.

#### Transfer Test Tasks

The layout of the *transfer test tasks* consisting of numerical- and formula tasks can be seen in Figures 2B,D. The rationale underlying why *transfer test tasks* constitute a more valid measure of exploring students’ conceptual understanding of mathematics is that the solution algorithm (e.g., *y* = 3*x* + 1) could have been memorized without any conceptual understanding. For a *transfer test tasks* the same algorithm cannot be used again, but the same general solution idea (e.g., multiplying the number of squares or rectangles with the number of matches needed for each new square/rectangle, and then adding the number of matches needed to complete the first square/rectangle) can be employed. We argue that knowing this idea of a general solution constitutes a local conceptual understanding of the task.

The Supplementary Material provides more examples of tasks.

### Practice and Test Settings

In all three experiments, the practice sessions and test sessions were conducted in the students’ classroom. Both sets of tasks were presented to the students on their laptops. All tasks were solved individually; hence, no teacher or peer support was provided. The students were offered the use of a simple virtual calculator, which was displayed on their laptop screen. After submitting each answer during a practice session, the correct answer was shown to the students. However, no correct answers were provided to tasks that asked the students to construct formulas (i.e., the third CMR task). This was done to prevent students from using a provided formula instead of constructing a method/formula.

The software that was used for presenting practice and test tasks also checked and saved the answers automatically. All students received the same elements of the intervention, which due to the computer presentations, were delivered in the same manner to all the students, ensuring high fidelity (Horner et al., 2006). The Supplementary Material provides additional examples and descriptions of the tasks employed in this study. The three experiments did not include a pre-test due to the risk of an interaction between the pre-test and the learning conditions, making the students more or less responsive to manipulation (for a discussion, see Pasnak, 2018). Moreover, the students were unfamiliar with the mathematical tasks.

### Cognitive Measurement

The cognitive measures included cognitive testing of a complex working memory task (operation span; Unsworth et al., 2005) and general fluid intelligence (Raven’s Advanced Progressive Matrices; Raven et al., 2003). Raven’s APM consists of 48 items, including 12 practice items. To capture individual differences and to prevent both ceiling and floor effects, we used the 12 practice items as well as the 36 original test items. The 12 practice items were validated against Raven’s Standard Progressive Matrices (Chiesi et al., 2012). These 48 test items were divided into 24 odd-numbered and 24 even-numbered items. Half of the students were randomly assigned to the odd-numbered items and half were assigned the even-numbered items. The total number of correct solutions was summed, providing a maximum score of 24. The task was self-paced over a maximum of 25 min. The countdown from 25 min was displayed in the upper-right corner of the screen. Initially, the students practiced on three items derived from Raven’s Standard Progressive Matrices. A measure of internal consistency (Cronbach’s alpha) was extracted from a larger pool of data, which encompassed the data obtained from the students in experiments 1 and 2, and was found to be 0.84.

In the operation span task students were asked to perform mathematical operations while retaining specific letters in their memory. After a sequence of mathematical operations and letters, they were asked to recall these letters in the same order as they were presented. The mathematical operations were self-paced (with an upper limit of 2.5 standard deviations above each individual average response time, extracted from an initial practice session). Each letter was presented after each mathematical operation and displayed for 800 ms. The letters to recall were presented in three sets of each set size. Every set size contained three to seven letters. The sum of all entirely recalled sets was used as the student’s WMC score. The measure of internal consistency revealed a Cronbach’s alpha of 0.83. Operation span was also self-paced, but without any time limit.

The operation span task and Raven’s matrices were combined into a composite score denoted as the cognitive proficiency (CP) index. The CP index score was based on a *z*-transformation of the operation span task performance and Raven’s matrices, thus forming the CP composite scores. These CP composite scores were then used to split (median split) students into lower and higher CP groups, and were used as a factor in the subsequent analyses across all three experiments. The students conducted the cognitive tests in their classrooms approximately 1 week before each of the three experiments.

### Experiment 1

#### Participants

*A priori* power analysis with effect sizes (*d* = 0.73) from Jonsson et al. (2014) indicated that with an alpha of 0.05 and a statistical power of 0.80, a sample size of 61 students would obtain a statistical group difference. The students attended a large upper secondary school located in a municipality in a northern region of Sweden. Recruitment of students was conducted in class by the authors. One hundred and forty-four students were included in the experiment. Within each math track (basic, advanced) students were randomly assigned to engage in either the AR or CMR^3^ groups. Out of those, 137 students (63 boys, 74 girls) with a mean age of 17.13 years (*SD* = 0.62) were included and subsequently analyzed according to their natural science (advanced level), social science (basic level) math tracks and CP. All students spoke Swedish. Written informed consent was obtained from the students in accordance with the Helsinki declaration. The Regional Ethics Committee at Umeå University, Sweden, approved the study.

#### Cognitive Measures

The cognitive testing included measures of the working memory task (operation span; Unsworth et al., 2005) and general fluid intelligence (Raven’s matrices; Raven et al., 2003). The mean value for the operation span task was 31.52 (*SD* = 16.35) and 12.63 (*SD* = 5.10) for Raven’s matrices, respectively. The correlation between the operation span and the Raven’s matrices was found to be significant, *r* = 0.42, *p* < 0.001. A CP composite score was formed based on the operation span and Raven’s matrices scores, and was used to split the students into low and high CP groups; it was also used as a factor in the subsequent analyses.

#### Tasks

From the 28 designed tasks (see above), 14 *practice tasks* were randomly chosen for the practice session. The corresponding 14 *practiced test tasks* together with seven *transfer test tasks* were used during the test.

#### Procedure

In a between-group design, the students engaged in either the AR practice (*N* = 72), which involved solving 14 AR task sets (Figure 1A), or the CMR (*N* = 65) practice, which involved solving 14 CMR task sets (Figure 1B). The students had 4 min to conclude each of the 14 task sets.

One week later, a test was conducted in which students were asked to solve 14 *practiced test tasks*, formula and numerical tasks (Figures 2A,C) and seven *transfer test tasks*, formula and numerical tasks (Figures 2B,D). The first test task for both the *practiced test tasks* and the *transfer test tasks* was to write down the formula corresponding to the *practice task* with a time limit of 30 s. The second test task for both the *practiced test tas*ks and the *transfer test tasks* was comprised of solving a numerical test task. The students were given 4 min to solve each task. The *practiced test tasks* were always presented before the *transfer test tasks*.

#### Statistical Analysis

A 2 (CP; low, high) × 2 (group; AR, CMR) × 2 (math tracks; basic, advanced) multivariate analysis of variance (MANOVA) was followed by univariate analyses of variance (ANOVAs). The proportions of correct responses on numerical (practiced, transfer) and formula (practiced, transfer) tasks were entered as the dependent variables. Cohens *d*, and partial eta square (η_*p*_^2^) were used as index of effect sizes.

## Results

Table 1A displays mean values, standard deviations, skewness, kurtosis, and Cronbach’s alpha of proportion correct responses for the test tasks for both AR and CMR learning conditions. Separate independent *t*-tests revealed that there were no significant differences between students in the AR and CMR learning conditions for operation span, *t*(135) = 0.48, *p* = 63, *d* = *0.08* and for the Raven’s matrices, *t*(135) = 0.12, *p* = 0.90, *d* = 0.02, respectively, showing that these groups were equal with respect to both complex working memory and fluid intelligence. Moreover, a subsequent analysis (independent *t*-test) of the CP composite score dividing the students into high and low CP groups showed that they could be considered to be cognitively separated, *t*(135) = 15.71, *p* < 0.001, *d* = 2.68.

**TABLE 1A:** Mean proportion correct response (*M*) and standard deviations (*SD*), skewness, kurtosis and Cronbach’s alpha for the AR and CMR learning conditions, respectively.

Learning condition (AR/CMR)	M	SD	Skewness	Kurtosis	Cronbach’s alpha
**AR**					
Practiced test task formula	0.08	0.13	2.13	3.50	0.86
Practiced test task numerical	0.26	0.26	0.95	0.25	0.83
Transfer test task formula	0.12	0.18	1.72	3.07	0.61
Transfer test task numerical	0.27	0.26	0.87	–0.11	0.71
**CMR**					
Practiced test task formula	0.20	0.22	0.97	–0.11	0.86
Practiced test task numerical	0.42	0.25	0.06	1.06	0.88
Transfer test task formula	0.19	0.22	1.40	1.58	0.62
Transfer test task numerical	0.35	0.30	0.59	–0.68	0.76

*AR, algorithmic reasoning, CMR, creative mathematical reasoning.*

Table 1B display proportion correct responses for the test tasks divided according to their CP level. The statistical analyses confirmed that the students in the CMR learning condition outperformed those in the AR learning condition, *F*(4,126) = 4.42, *p* = 0.002, Wilk’s Λ = 0.40, η_*p*_^2^ = 0.12. Follow-up ANOVAs for each dependent variable were significant, *practiced test task formula*, *F*(1,129) = 15.83, *p* < 0.001, η_*p*_^2^ = 0.10; *practiced test task numerical*, *F*(1,129) = 12.35, *p* = 0.001, η_*p*_^2^ = 0.09; *transfer test task formula*, *F*(1,129) = 8.83, *p* = 0.04, η_*p*_^2^ = 0.06; and *transfer test task numerical*, *F*(1,129) = 5.05, *p* = 0.03, η_*p*_^2^ = 0.04. An effect of CP was also obtained, *F*(4,126) = 7.71, *p* < 0.001, Wilk’s Λ = 0.80, η_*p*_^2^ = 0.20, showing that the more cognitively proficient students outperformed those who were less proficient. Follow-up ANOVAs for each dependent variable revealed significant univariate effects of CP for the *practiced test task formula*, *F*(1,129) = 12.35, *p* < 0.001, η_*p*_^2^ = 0.09; the *practiced test task numerical*, *F*(1,129) = 25.72, *p* < 0.001, η_*p*_^2^ = 0.17; the *transfer test task* formula, *F*(1,129) = 22.63, *p* < 0.001, η_*p*_^2^ = 0.15; and the *transfer test task numerical*, *F*(1,129) = 22.46, *p* < 0.01, η_*p*_^2^ = 0.15. However, no multivariate main effects of math tracks and no multivariate interactions were obtained, with all *p’s* > 0.10.

**TABLE 1B:** Mean proportion correct response (*M*) and standard deviations (*SD*) for AR and CMR learning conditions across low and high CP groups.

CP group (low/high)	*AR*		*CMR*	
	*M*	*SD*	*M*	*SD*
**Low CP^1^**				
Practiced test task formula	0.04	0.11	0.11	0.15
Practiced test task numerical	0.14	0.19	0.29	0.21
Transfer test task formula	0.05	0.10	0.08	0.14
Transfer test task Numerical	0.13	0.16	0.22	0.27
**High CP^2^**				
Practiced test task formula	0.11	0.15	0.29	0.24
Practiced test task numerical	0.38	0.26	0.54	0.23
Transfer test task formula	0.19	0.21	0.30	0.25
Transfer test task Numerical	0.41	0.27	0.48	0.27

*CP, cognitive proficiency, AR, algorithmic reasoning. CMR, creative mathematical reasoning. ^1^*n* = 69. ^2^*n* = 68.*

## Discussion

With respect to all four dependent variables, the analyses showed that students practicing with CMR had superior results on the subsequent test 1 week later than students practicing with AR (confirming hypotheses 1 and 2) and that the more cognitively proficient students outperformed their less cognitively proficient counterparts, independent of group (confirming hypothesis 3). Although the natural science students performed, on average, better than social science students on all four dependent variables, no significant main effect was observed for math tracks (disconfirming hypothesis 4).

### Experiment 2

The same hypotheses as in experiment 1 were posed in experiment 2. However, as pointed out above, there is a higher risk of non-equivalent group bias when using a between-subject design, and a simpler test format could challenge the differential effects found in experiment 1 (CMR > AR). It was therefore decided that experiment 2 should employ a within-subject design and use MC questions as a test format instead of short answers.

#### Participants

*A priori* power analysis based on a within-subjects pilot study (*N* = 20) indicated that with an alpha of 0.05 and a statistical power of 0.80, a sample size of 50 students would obtain a statistical group difference. The students were from a larger pool of students, of which 82 students were randomly allocated to a functional Magnetic Resonance Imaging (fMRI) study, and the remaining 51 students participated in experiment 2. An independent *t*-test revealed no differences concerning age, general fluid intelligence (Raven’s matrices), or WMC (operation span), with *p*-values > 0.37. The separate fMRI experiment is not reported here. Experiment 2 included 51 students (27 girls, 24 boys) from natural science and social science programs in three upper secondary schools located in a municipality in a northern region of Sweden with a mean age of 18.13 years (*SD* = 0.24). Recruitment of students was conducted in class by the authors, at each school. The natural science students were enrolled in more advanced math track compared with the Social science students; as in experiment 1, math tracks (basic, advanced) were subsequently entered as a factor in the analyses.

#### Cognitive Measures

As in experiment 1, the cognitive testing included operation span and Raven’s matrices. The mean values and standard deviations of the operation span and Raven’s matrices were similar to those in experiment 1, for the operation span task (*M* = 38.27, *SD* = 19.10) and Ravens matrices (*M* = 14.47, *SD* = 5.34), respectively. The correlation between operation span and Raven’s matrices was found to be significant, with *r* = 0.52 and *p* < 0.001. A CP composite score was formed based on the operation span and Raven’s matrices scores. The CP score was used to split the students into a low CP group and a high CP group, and was also used as a factor in the subsequent analyses.

#### Tasks

In a within-subject design, each student practice with 12 AR task sets and 12 CMR task sets. The corresponding 24 *practice test tasks*, together with eight *transfer test tasks*, were used as test tasks.

#### Procedure

In this within-subject design, the students first practiced with 12 AR task sets. After a break of a few hours, they then practiced with 12 CMR task sets. This order was chosen to avoid carry-over effects from CMR tasks to AR tasks. The rationale was that starting with CMR tasks would reveal the underlying manipulation, which the students could then use to solve the AR tasks. Hence, constructing the solution without using the provided formula is the critical factor in the manipulation. To prevent item effects in which some tasks were more suitable to be designed as AR or CMR tasks, the tasks that were, respectively, assigned to be CMR and AR tasks were counterbalanced. The students were given 4 min to conclude each of the 12 task sets.

One week later, the students were asked to solve 24 randomly presented *practiced test tasks* (albeit with different numbers than before), of which 12 had been practiced as CMR tasks and 12 as AR tasks. These tasks were followed by eight *transfer test tasks*.

#### Statistical Analyses

A mixed-design ANOVA was conducted with learning condition (AR and CMR) and task type (practiced and transfer) as the within-subject factors and CP (low and high) and math tracks (basic and advanced) as the between-subject factors. The proportions of correct responses on *practiced test tasks* and *transfer test tasks* were entered as the dependent variables. Cohens *d* and partial eta square (η_*p*_^2^) were used as index of effect sizes. Although a within-subject design was used, the more cognitively proficient students, who are likely to have better metacognitive ability (see Desoete and De Craene, 2019 for an overview), could potentially make use of constructive matching by comparing a possible solution with the response alternatives, response elimination by determining which answer is more likely, or of guessing (Arendasy and Sommer, 2013; see also Gonthier and Roulin, 2020). Therefore, the analysis was corrected using the formula FS = R – W/C – 1, where FS = formula score; R = number of items/questions answered correctly; W = number of items/questions answered incorrectly; and C = number of choices per item/question (e.g., Diamond and Evans, 1973; Stenlund et al., 2014).

## Results

Table 2A displays the mean values of proportion correct response (not corrected for guessing), standard errors, and psychometric properties of skewness, kurtosis, and Cronbach’s alpha for the test tasks. An independent *t*-test of the CP composite score dividing the students into high CP groups and low CP groups showed that the students could be considered to be cognitively separated, *t*(49) = 12.14, *p* < 0.001. *d* = 3.40. The table shows that the mean values for CMR are higher than the corresponding values for AR learning condition for both the *practiced test tasks* and *transfer test tasks*. Table 2B display proportion correct responses (not corrected for guessing) for the test tasks divided according to their CP level. The statistical analysis corrected for guessing revealed significant within-subject effects of learning condition, with the CMR condition being superior to the AR condition, *F*(1,47) = 9.36, *p* = 0.004, Wilk’s Λ = 0.83, η_*p*_^2^ = 0.17. However, there was no significant within-subject effect of task type, *F*(1,47) = 0.77, *p* = 0.38, Wilk’s Λ = 0.98, η_*p*_^2^ = 0.012. Moreover, there was no significant between-subject effects of CP, *F*(1,47) = 0.23, *p* = 0.64, η_*p*_^2^ = 0.004, or of math tracks, *F*(1,47) = 0.84, *p* = 0.36, η_*p*_^22^ = 0.005, and there were no interaction effects (*p’s* > 0.67).

**TABLE 2A:** Mean proportion correct response (*M*) and standard deviations (*SD*), skewness, kurtosis and Cronbach’s alpha for the AR and CMR learning conditions, respectively.

Learning condition (AR/CMR)	M	SD	Skewness	Kurtosis	Cronbach’s alpha
**AR**					
Practiced test task	0.48	0.25	0.27	−0.84	0.80
Transfer test task	0.46	0.33	0.35	−0.99	0.69
**CMR**					
Practiced test task	0.55	0.27	0.00	−1.40	0.82
Transfer test task	0.53	0.35	0.00	1.40	0.63

*AR, algorithmic reasoning, CMR, creative mathematical reasoning.*

**TABLE 2B:** Mean proportion correct response (*M*) and standard deviations (*SD*) for AR and CMR learning conditions across low and high CP groups.

CP group (low/high)	AR		CMR	
	*M*	*SD*	*M*	*SD*
**Low CP^1^**				
Practiced test task	0.35	0.17	0.40	0.21
Transfer test task	0.33	0.28	0.29	0.22
**High CP^2^**				
Practiced test task	0.61	0.24	0.76	0.24
Transfer test task	0.58	0.33	0.69	0.30

*CP, cognitive proficiency, AR, algorithmic reasoning. CMR, creative mathematical reasoning. ^1^*n* = 25 ^2^*n* = 26*

The non-significant effect of CP was rather surprising; therefore, it was decided to re-run the analyses without the correction formula. The analyses again revealed a significant within-subject effect of learning condition, with the CMR condition being superior to the AR condition, *F*(1,47) = 7.80, *p* = 0.008, Wilk’s Λ = 0.85, η_*p*_^2^ = 0.14. Again no significant within-subject effects from task type, *F*(1,47) = 2.3, *p* = 0.13, Wilk’s Λ = 0.95, η_*p*_^2^ = 0.02 or between-subject effect of math tracks, *F*(1,47) = 3.45, *p* = 0.07, η_*p*_^2^ = 0.07 were detected. However, the between-subject effect of CP was now clearly significant, *F*(1,47) = 18.74, *p* < 0.001, η_*p*_^2^ = 0.28. Moreover, a learning condition × CP interaction *F*(1,47) = 9.05, *p* = 0.004, Wilk’s Λ = 0.83 η_*p*_^2^ = 0.16 was qualified by a learning condition × task type × CP interaction, *F*(1,47) = 8.10, *p* = 0.005, Wilk’s Λ = 0.84, η_*p*_^2^ = 0.16. The three-way interaction was driven by students with a high CP performing better in the CMR learning condition than in the AR learning condition especially pronounced for the transfer test tasks. No other interaction effects were detected (*p’s* > 0.70).

## Discussion

With respect to both *practiced test tasks* and *transfer test tasks*, the analyses showed, as expected, that students who practiced with CMR had superior results on the subsequent tests 1 week later compared to the students who practiced with AR (confirming hypothesis 1 and 2). In comparison with experiment 1, experiment 2 showed notably higher performance levels, which most likely reflected the MC test format. Viewed in relation to previous studies of CMR (e.g., Jonsson et al., 2014) and the significant number of studies showing that educational attainments in math are intimately related to cognitive abilities (e.g., Adam and Hitch, 1997; Andersson and Lyxell, 2007), the non-significant effect of CP was unexpected. The finding that task type was non-significant, albeit in the direction of the *practiced test task* being easier than the *transfer test tasks* was also somewhat unexpected. It is possible that the eight *transfer test tasks* (four AR and four CMR) may have been too few to build reliable statistics. Although no significant effect was obtained for math tracks, the natural science students (advanced math track) performed better than the social science students (basic math track) on average; however, this trend did not reach statistical significance (disconfirming hypothesis 4). After the unexpected non-significant effect of CP, the analysis was re-run without the correction formula. The analysis revealed a main effect of CP (confirming hypothesis 3) and a learning condition × CP interaction that was qualified by a learning condition × task type × CP interaction. The three-way interaction indicates that cognitively stronger students could utilize response elimination or successful guessing in subsequent MC tests more effectively than their lower CP counterparts, especially for the transfer test tasks.

This design, in which the CMR practice tasks were presented shortly after the AR tasks, may have introduced a recency effect and thus facilitated the test performance more for CMR than for AR tasks. However, the CMR practice session contained 12 different task sets, and each new task set was a potential distractor for the previous task sets. Moreover, between the learning session and subsequent test 1 week later, the students attended their regular classes. These activities, viewed in conjunction with the well-known fact that the recency effect is rather transitory (Koppenaal and Glanzer, 1990) and that recall is severely disrupted even by unrelated in-between cognitive activities (Glanzer and Cunitz, 1966; Kuhn et al., 2018), probably eliminated the risk of recency effects. In experiment 2, the total number of test tasks was 32 (24 *practiced test tasks* and eight *transfer test tasks*), and some students complained that there were too many tasks, which may have affected their performance, potentially the cognitively more proficient students were less affected by the large number test tasks.

### Experiment 3

In experiment 3, the same hypotheses were posed as in experiments 1 and 2. However, as pointed out above, the more cognitively proficient students were potentially less affected by fatigue and gained more from using MC questions as a test format. Therefore, it was decided that experiment 3 should retain the within-subject design but use only *transfer test tasks*. Moreover, we reintroduced written answers as a response mode to prevent processes of constructive matching and response elimination. To reduce a potential, but unlikely, recency effect, the presentation order for a subsample was reversed, with CMR tasks being presented before AR tasks.

#### Participants

Experiment 3 included 82 students. The average age of participants was 17.35 years (*SD* = 0.66), whereof 35 were girls, and 47 boys. The participants were from two upper secondary schools located in a municipality in a northern region of Sweden. Recruitment of students was conducted in class by the authors, at each school. The students were divided into two math tracks. The first was a mathematical track that included year 3 technical students and year 2 natural science students (advanced math tracks); these students were regarded by their schoolteachers as approximately equal in math skill background. The second math track consisted of year 1 natural science students and year 2 social science students (basic math track); these students were also regarded as approximately equal in math skill background. The students were subsequently analyzed according to their math tracks.

#### Cognitive Measures

The cognitive tests were the same as in experiments 1 and 2. The mean values and standard deviations of the operation span (*M* = 36.78, *SD* = 16.07) and Raven’s matrices (*M* = 14.33, *SD* = 4.35) were similar to those from experiments 1 and 2. The correlation between operation span and Raven’s matrices was found to be significant, with *r* = 0.40 and *p* < 0.001, and a CP composite score based on operation span and Raven’s matrices scores was again formed, used to split the students into a low CP group and a high CP group, and used as a factor in the subsequent analyses.

#### Tasks

The same practice tasks were used, as in experiment 2. In a within-subject design, the students practiced with 12 AR task sets and 12 CMR task sets, and 24 *transfer test tasks* were used during the test.

#### Procedure

The students practiced with the same tasks and setup as in experiment 2, with the exception that the order of presentation was reversed for a subset of students, with AR tasks being practiced before CMR tasks. The students had 4 min to conclude each of the 12 task sets during practice. One week later, the students were asked to solve 24 *transfer test tasks*. The students were given 130 s to solve each test task.

#### Statistical Analyses

The initial mixed-design ANOVA analysis, with learning condition (AR and CMR) as the within-subject factor and order of presentation as the between-subject variable and the proportion correct response as the dependent variable, investigated the potential presentation order × learning condition interaction. The analysis revealed that this interaction was non-significant, with *F*(1,80) = 0.22, *p* = 0.88, Wilk’s Λ = 0.10, η_*p*_^2^ = 0.0004. Therefore, the presentation order was excluded from further analyses. Considering that the students differed in age (by approximately 1 year), we controlled for age by conducting a mixed-design analysis of covariance (ANCOVA) with learning condition (AR and CMR) as a within-subject factor and with CP (low and high) and math track (basic and advanced) as the between-subject factors. The proportion of correct responses on the *transfer test tasks* was entered as the dependent variable, and age was used as a covariate. Cohens *d* and partial eta square (η_*p*_^2^) were used as index of effect sizes.

## Results

Table 3A displays the mean values, standard deviations, skewness, kurtosis, and Cronbach’s alpha for the test tasks. An independent *t*-test of the CP composite score used to divide the students into a high CP group and a low CP group showed that the students could be considered as cognitively separated, *t*(80) = 12.88, *p* < 0.001, *d* = 2.84. The table shows that practicing with the CMR tasks was superior to practicing with the AR tasks. Table 3B display proportion correct responses for the transfer test tasks divided according to their CP level. The statistical analysis confirmed a within-subject effect of learning condition, *F*(1,77) = 20.88, *p* < 0.001, Wilk’s Λ = 0.78, η_*p*_^2^ = 0.21. The analysis also revealed a between-subject effect of CP, *F*(1,76) = 21.50, *p* < 0.001, η_*p*_^2^ = 0.22. However, no between-subject effect of math tracks and no interaction effects were obtained, *p’s* > 0.15.

**TABLE 3A:** Mean proportion correct response (*M*) and standard deviations (*SD*), skewness, kurtosis and Cronbach’s alpha for the AR and CMR learning conditions, respectively.

Learning condition (AR/CMR)	M	SD	Skewness	Kurtosis	Cronbach’s alpha
**AR**					
Transfer test task	0.26	0.21	0.78	−0.12	0.78
**CMR**					
Transfer test task	0.36	0.23	0.37	−0.33	0.75

*AR = Algorithmic Reasoning, CMR = Creative Mathematical Reasoning.*

**TABLE 3B:** Mean proportion correct response (*M*) and standard deviations (*SD*) for AR and CMR learning conditions across low and high CP groups.

CP group (low/high)	AR		CMR	
	*M*	*SD*	*M*	*SD*
**Low CP^1^**				
Transfer test task	0.19	0.15	0.26	0.18
**High CP^2^**				
Transfer test task	0.34	0.24	0.47	0.22

*CP, cognitive proficiency; AR, algorithmic reasoning; CMR, creative mathematical reasoning. ^1^*n* = 41. ^2^*n* = 41.*

## Discussion

The findings from experiment 3 were in line with those from the previous experiments, providing evidence that practicing with CMR tasks was superior to practicing with AR tasks (confirming hypotheses 1 and 2). As expected, the analyses showed that the more cognitively proficient students outperformed those who were less cognitively proficient (confirming hypothesis 3). Again, no significant effect was obtained for math tracks (again disconfirming hypothesis 4).

## General Discussion

This study contrasted CMR with AR across three experiments encompassing 270 students. It was hypothesized that practicing with CMR leads to better performances than practicing with AR on *practiced test tasks* and *transfer test tasks* (hypotheses 1 and 2). Experiments 1 and 2 included both *practiced test tasks* and *transfer test tasks*, while experiment 3 focused exclusively on *transfer test tasks*. The *practiced test tasks* were identical to the tasks that the students had practiced (albeit with different numbers). The *transfer test tasks* were different from the *practice tasks*, but they shared an underlying solution idea. To solve the *transfer test tasks*, the students had to rely on relevant knowledge (a solution idea) acquired during their practice, which is critical in mathematics. If a student has no solution idea to rely on, the *transfer test tasks* required the student to construct the method from scratch.

Moreover, this study hypothesized that the more cognitively proficient students would outperform those who were less cognitively proficient (hypothesis 3), independent of learning conditions. The upper secondary students were from different student programs with different mathematical backgrounds (i.e., basic and advanced math tracks), which was entered as a factor in the analyses. It was expected that those enrolled in a more advanced math track would outperform those enrolled in a basic math track (hypothesis 4).

Overall, the results confirmed hypotheses 1–3. However, no effects of math tracks were obtained, disconfirming hypothesis 4. Below, these hypotheses are discussed in detail.

### Hypotheses 1 and 2

The analysis of both the *practiced test tasks* in experiment 1 followed the setup of Jonsson et al. (2014), in which the dependent variables included trying to remember specific formulas and solving numerical *practiced test tasks*. Moreover, experiment 1 also went beyond Jonsson et al. (2014) and added *transfer test tasks*. The results of experiment 1 were in line with those of Jonsson et al. (2014): Practicing with CMR tasks lead to significantly better performance on the *practiced test tasks* than practicing with AR tasks. Experiment 1 also found that practicing with CMR lead to significantly better performance on *transfer test tasks*. In experiment 2, we turned to a within-subject design, with the aim of removing potential non-equivalent group bias, and introduced MC questions as a test format, thereby challenging hypotheses 1 and 2 by using an easier test format. Again, significant CMR > AR effects were detected for both *practiced test tasks* and *transfer test tasks*. However, the fact that only four AR and four CMR *transfer test tasks* were used in experiment 2, the results could be questioned in terms of building adequate statistics. Therefore, using a within-subject design, experiment 3 focused solely on *transfer test tasks*, which increased the number of *transfer test tasks* and reduced the total number of tasks and, thus, the risk of fatigue. We also reintroduced written answers as a response mode to prevent processes of response elimination and guessing. The analysis of experiment 3 revealed that practicing with CMR tasks had a more beneficial effect than practicing with AR tasks on the *transfer test tasks*, again confirming hypothesis 2.

### Hypothesis 3

When a short answer format was used, as in experiments 1 and 3, the effects of CP were clear, confirming previous studies and hypothesis 3. The second analysis in experiment 2 also confirmed hypothesis 3. The analysis showed that all participants improved their performance; hence the proportion correct was higher in experiment 2 than in experiments 1 and 3 (Tables 1–3). This performance was most likely due to the MC response mode. The second analysis indicates that the cognitively more proficient students could in addition, use response elimination or successful guessing more effective (Desoete and De Craene, 2019), thereby outperforming the cognitively less proficient. However, when the analysis was corrected for guessing (the first analysis), the benefits of using response elimination or guessing were removed, but the effects of the easier MC response mode remained, which even out the difference between the CP groups and thereby also removed the effect of CP.

### Hypothesis 4

The non-significant effect of mathematical track was somewhat surprising, and disconfirmed hypothesis 4. A plausible interpretation is that the students enrolled in more advanced math tracks, which involve (according to the curriculum) better mathematical training, could not make use of their acquired mathematical knowledge when solving the novel experimental test tasks; if this interpretation is correct, it would indicate that the assumption of task novelty was also correct.

Overall, this study provides support for the argument that CMR facilitates learning to a greater degree than AR and confirms the results of previous studies (Jonsson et al., 2014, 2016; Norqvist et al., 2019). Although the effect sizes were rather small, they must be viewed in relation to the short interventions that the students went through. We argue that when students are practicing with CMR tasks, they are “forced” to pay attention to the intrinsic and relevant mathematical components, which develops their conceptual understanding. The effects on *transfer test tasks* indicate that practicing with CMR tasks—in comparison with practicing with AR tasks—facilitates students’ ability to transfer their knowledge to a greater extent; that is, they can better transfer their solution idea from the practice task to a different task sharing the same underlying solution idea *(transfer test tasks*). This argument is in line with the findings of the Norqvist et al. (2019) eye-tracking study: When students practiced with AR tasks, they disregarded critical information that could be used to build a more in-depth understanding; in contrast, students that practiced with CMR tasks focused on critical information more frequently. Practice with CMR is most likely associated with more effortful struggle—an argument that shares similarities with the framework of “ill-structured tasks” (Kapur, 2008, 2010). In the ill-structured task approach, students are provided with tasks for which no method or procedure on how to solve the task is available and for which multiple solution paths may exist. Students are required to (try to) solve the ill-structured task by constructing their own methods before the teacher provides instructions on the mathematics to be learned (VanLehn et al., 2003; Kapur, 2010). Those studies showed that the struggle of creating methods was especially beneficial for developing a conceptual understanding of the task, as demonstrated by significantly better performance on *transfer test tasks* (e.g., Kapur, 2010, 2011). It is argued that the task complexity inherent in the ill-defined tasks was a key factor that helped students to create structures that facilitated their conceptual understanding of mathematics. Furthermore, studies have shown that the more solutions students generate on their own, the better the students’ test performance becomes, even when their methods do not fully solve the practice task (Kapur, 2014). In the CMR tasks used in the present study, no instructions were given. Similar to the ill-structured approach, such tasks may identify knowledge gaps and enable (or “force”) students to search for and perceive in-depth structural problem features (Newman and DeCaro, 2019). Although an excessively high cognitive load may hamper learning, a desirable amount of cognitive load in terms of struggle (in a positive sense) with mathematics may be beneficial for developing conceptual understanding (Hiebert and Grouws, 2007). In the present study, such development of students’ conceptual understanding was seen in the form of better performance on the later test as a function of practicing with CMR tasks relative to AR tasks.

This study provides support for the theoretical link between the learning process using CMR, performance, and conceptual understanding. The results also underscore that although CP was associated with better performance, it did not interact with the learning condition. Hence, both cognitively stronger and cognitively weaker students benefited from using CMR relative to using AR. The theoretical framework (Lithner, 2008, 2017) could potentially be updated with an individual differences perspective with respect to cognitive prerequisites and their implication for the learning process. With respect to the non-significant effect of math tracks, the assumption of task novelty seems to be correct. Moreover, the non-significant effect of math tracks also indicates that students can gain conceptual understanding by using CMR even with tasks for which the students lack or have negligible pre-knowledge, and among students with “only” basic mathematical background.

The results from this study could be discussed from a self-explanation perspective (for an overview, see Rittle-Johnson et al., 2017). According to Rittle-Johnson et al. (2017), the mechanism underlying self-explanation is the integration of new information with previous knowledge. This involves guiding students’ attention to the structural features—rather than the surface features—of the to-be-learned material, and can aid comprehension and transfer. In the CMR assumption, predictive arguments supporting strategy choice and verification arguments explaining why the strategy implementation and conclusions are “true or plausible” are regarded as critical features.

In sum, in the CMR/AR, ill-structured tasks, and self-explanation approaches, the critical aspects are how tasks are designed and how mathematical reasoning is supported. Moreover, in order to move beyond textbooks’ step-by-step solutions and understand the underlying ideas, students need to face (in a positive sense) mathematical struggle activities. Nevertheless, it is not likely that students will take on such effort by themselves. The framework of CMR and ill-structured tasks removes the task-solving methods and requires students to find an underlying idea and to create solutions on their own. Although CMR task solving is more cognitively demanding during practice than AR task solving, it helps the learner to focus on relevant information for solving the task. Moreover, similar to the self-explanation approach, the CMR approach guides students to the structural features that are critical for aiding comprehension.

### Limitations

A limitation in the present study is that experiment 3 did not include any *practiced test tasks*. However, the results from experiments 1 and 2 indicate that using *practiced test tasks* in experiment 3 would have yielded the same conclusions as in experiments 1 and 2. A further potential limitation is that the presentation format differed in experiment 2 in comparison with experiments 1 and 3. However, it could in fact be argued that this is a strength of the study: Despite the different response formats for the test tasks, the experiments yielded similar results, with CMR consistently outperforming AR. Although the experiments were based on convenience samples, which could potentially narrow the external validity, the students were from four different upper secondary schools, which provided some heterogeneity. The results can also be discussed from the perspective of Hawthorne effects: The awareness of knowing that they were part of a study may have affected the students’ performance, and—although this is unlikely—the findings may not generalize to a regular setting when the researcher is not present.

Moreover, there were no pre-test measures in any of the experiments, as it was argued that a pre-test could make the students more or less responsive to the manipulation (see Pasnak, 2018, for a discussion). On the other hand, pre-tests could have provided insight into how comprehension increased from the pre- to a post-test. In experiment 1, pre-tests would have provided a baseline of student performance, which could have been used to evaluate initial group differences.

### Implications and Future Research

The results from the present and previous studies (e.g., Jonsson et al., 2014, 2016; Norqvist et al., 2019) have implications for school settings, as AR tasks (as opposed to CMR tasks) are commonly used in teaching approaches and textbooks (Stacey and Vincent, 2009; Denisse et al., 2012; Shield and Dole, 2013; Boesen et al., 2014; Jäder et al., 2019), but as argued do not promote optimal student learning. We argue that an eclectic perspective in which validated methods that emphasize mathematical struggles—such as task solving using CMR, ill-structured tasks, and self-explanations—should be a part of the mathematical curriculum, in conjunction with approaches that reduce cognitive load, such as worked examples. In future studies, it would be interesting not only to contrast CMR with other approaches, but also to investigate how to combine the CMR approach with, for example, self-explanation (Rittle-Johnson et al., 2017) and, potentially, with worked examples as well (Sweller et al., 2011). Another potential combination could involve retrieval practice, which is a cognitive-based learning strategy based on self-testing. At first glance, retrieval practice is very different from using CMR. Using CMR emphasizes the construction of solutions, while retrieval practice strengthens memory consolidation through the process of retrieving information from long-term memory. For example, retrieving the definition of working memory without the support of written text will enhance one’s ability to remember the definition across long-term retention intervals (Wiklund-Hörnqvist et al., 2014). The performance difference between retrieval practice and other ways of attaining information—most commonly re-reading—is denoted as the “testing effect.” The testing effect is supported by both behavioral and functional fMRI evidence (for overviews, see Dunlosky et al., 2013; van den Broek et al., 2016; Adesope et al., 2017; Antony et al., 2017; Moreira et al., 2019; Jonsson et al., 2020). Research that currently underway shows that measures of brain activity following the testing effect (retrieval practice > study) and the “CMR effect” (CMR > AR) indicate that the same brain areas are activated. It is possible that by adding retrieval practice after formulas or procedures have been established by using CMR, the memory strength of specific formulas may be enhanced. Future studies are planned to pursue this reasoning.

Moreover, as stated in the limitation, the experiments in the present study were based on convenience samples. A purely randomized sampling or a stratified sampling would be preferable in future studies. It is also unclear whether the CMR approach is potent among students with special needs, although the non-significant effects of math tracks found in the present study were encouraging; future studies should pursue this question.

## Data Availability Statement

The datasets are available on request to the corresponding author.

## Ethics Statement

The studies involving human participants were reviewed and approved by The Regional Ethics Committee at Umeå University, Sweden approved the study at University. Written informed consent from the participants’ legal guardian/next of kin was not required to participate in this study in accordance with the national legislation and the institutional requirements.

## Author Contributions

BJ, CG, and JL came up with the idea for the study and jointly contributed to the conceptualization and design of the study and revised the manuscript for important intellectual content. CG and BJ conducted the data collection. BJ performed the statistical analysis and wrote the first draft of the manuscript. All authors contributed to manuscript revision and read and approved the submitted version.

## Conflict of Interest

The authors declare that the research was conducted in the absence of any commercial or financial relationships that could be construed as a potential conflict of interest.

## Footnotes

1 https://www.nctm.org/26T

2 www.skolverket.se

3 In the CMR group data from six participants were lost due to administrative error.
