# Supplementary material for: Gaining Mathematical Understanding: The Effects of Creative Mathematical Reasoning and Cognitive Proficiency
Source: Front Psychol. 2020 Dec 18;11:574366. doi: 10.3389/fpsyg.2020.574366 (PMC7775304; doi:10.3389/fpsyg.2020.574366)
Supplement: Supplementary file 1 [file Data_Sheet_1.PDF]

A) AR-practice task

When squares are put in a row, it looks like the figure on the right, 13 matches are needed for four squares.

If x is the number of squares then the number of matches y could be calculated by the function **$y = 3x + 1$**

Example: If 4 squares are put in a row then $y = 3x + 1 = 3\cdot4+1 = 13$ matches are needed

How many matches are needed to get 100 squares in a row?

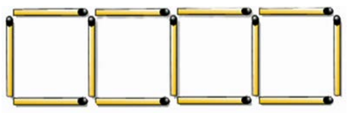

B) CMR-practice task

When squares are put in a row, it looks like the figure on the right, 13 matches are needed for four squares.

How many matches are needed to get 100 squares in a row?

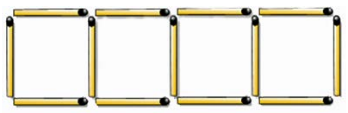

C) Transfer test task

When rectangles are put in a row, it looks like the figure on the right, 15 matches are needed for three rectangles.

How many matches are needed to get 100 rectangles in a row?

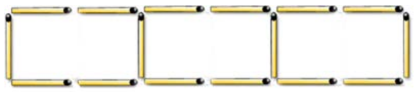

D) AR-practice task

With 6 matches, you can build a house. The houses can be put together into a row of houses. The outer edge is built of red matches

If x is the number of houses, the number of red matches y can be calculated by the formula **$y = 3x + 2$**

Example: If 4 houses are put together, $y = 3 \cdot 4 + 2 = 14$ red matches are needed

How many red matches are needed to put 100 houses together?

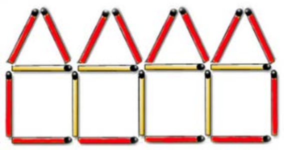

E) CMR-practice task

With 6 matches, you can build a house. The houses can be put together into a row of houses. The outer edge is built of red matches. If 4 houses are put together in a row, 14 red matches are needed

How many red matches are needed to put 100 houses together?

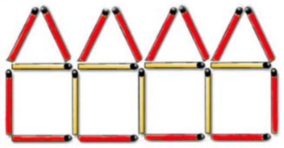

F) Transfer test task

With 8 matches, you can build a two-stored house. The houses can be put together into a row of houses. The outer edge is built of red matches. If 4 two-stored houses are put together in a row, 14 red matches are needed

How many red matches are needed to put 100 two-stored houses together?

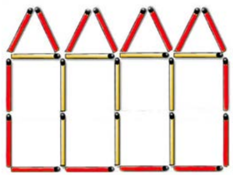

G) AR-practice task

A blue string, as in the figure, is divided into pieces by cuts along the red lines

If you cut x times, the number of pieces y can be calculated by the formula
y = 3x +1

Example: If 4 cuts are made, the string is divided into y = 3 · 4 + 1 = 13 pieces

How many pieces do you get if 40 cuts are made?

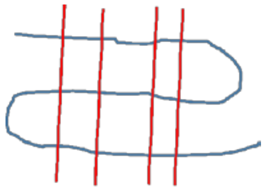

H) CMR-practice task

A blue string, as in the figure, is divided into pieces by cuts along the red lines.

If 4 cuts are made, the string is divided into 13 pieces

How many pieces do you get if 40 cuts are made?

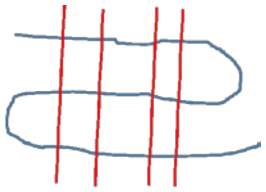

I) Transfer test task

A blue string, as in the figure, is divided into pieces by cuts along the red lines.

If 4 cuts are made, the string is divided into 17 pieces

How many pieces do you get if 40 cuts are made?

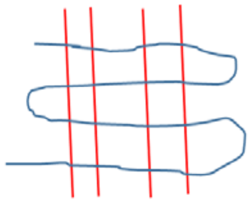

J) AR-practice task

An area that is a dm · b dm has been tiled. The tiles shaped as squares have an edge length of 1 dm. Inside a frame with white tiles, there are red tiles.
If the area is a dm · b dm, the number of red tiles y can be calculated by the formula
y = ab-2a-2b + 4
Example: If the area is 6 dm · 8 dm then there are y = 6·8 - 2·6 - 2·8+4 = 24 red tiles

How many RED tiles are needed if the area to be tiled is 59 dm · 71 dm?

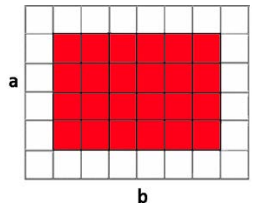

K) CMR-practice task

An area that is a dm · b dm has been tiled. The tiles shaped as squares have an edge length of 1 dm. Inside a frame with white tiles, there are red tiles.
If the area is 6 dm · 8 dm then 24 red tiles are needed

How many RED tiles are needed if the area to be tiled is 59 dm · 71 dm?

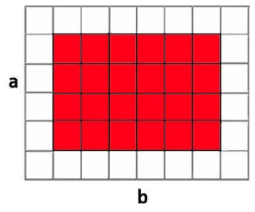

L) Transfer test task

An area that is a dm · b dm has been tiled. The tiles shaped as squares have an edge length of 1 dm. Inside a frame with two rows of white tiles, there are red tiles. If the area is 8 dm · 10 dm then 24 red tiles are needed

How many red tiles are needed if the area to be tiled is 68 dm · 92 dm?

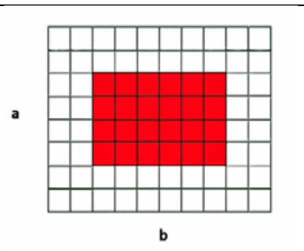

M) AR-practice task

A tower is built of tiles that are 1 cm thick and rods that are 10 cm long.
The tiles are used for floors and ceilings and the rods for the walls.
If you have x rods, the height of the tower, y (assuming the plates are sufficient) can be calculated by the formula **y = 2.75x + 1**

Example: If we have 8 rods (and enough tiles) then the tower will be
y = 2.75 · 8 + 1 = 23 cm high

How tall will the tower be if you build with 100 rods?

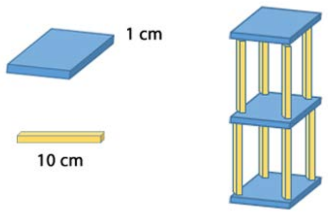

N) CMR-practice task

A tower is built of tiles that are 1 cm thick and rods that are 10 cm long.
The tiles are used for floors and ceilings and the rods for the walls.
If we have 8 rods (and enough tiles), then the tower will be 23 cm high

How tall will the tower be if you build with 100 rods?

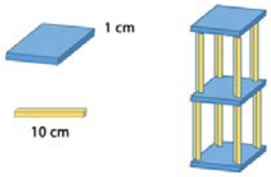

O) Transfer test task

A tower is built of tiles that are 1 cm thick and rods that are 10 cm long.
The tiles are used for floors and ceilings and the rods for the walls.
If we have 6 rods (and enough tiles), then the tower will be 23 cm high

How tall will the tower be if you build with 300 rods?

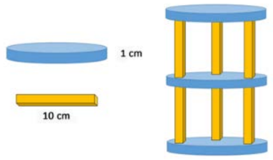

P) AR-practice task

A regular chessboard has 8 · 8 squares, 32 black and 32 white squares.
If the chessboard has x · x squares, and there are black squares in all corners, the number of black squares y can be calculated by the formula $\mathbf{y = 0.5x^2 + 0.5}$

Example: If there are 5·5 squares, and black squares in the corners, then there are >
$y = 0.5 \cdot 5^2 + 0.5 = 13$ black squares

How many black squares has a chessboard, that has black squares in the corners and is 51 · 51 squares large?

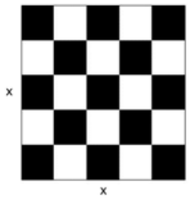

Q) CMR-practice task

A regular chessboard has 8 · 8 squares, 32 black and 32 white squares.
If the chessboard has 5·5 squares and there are black squares in all corners, then the chessboard has 13 black squares

If the chessboard has 51 · 51 squares and there are black squares in the corners how many of the squares are black?

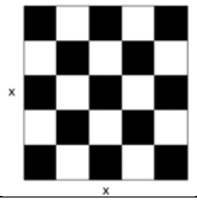

R) Transfer test task

A regular chessboard has 8 · 8 squares, 32 black and 32 white squares.
If the chessboard has·5   squares and there are black squares in all corners, then the chessboard has 12 white squares

If the chessboard has 51 · 51 squares and there are black squares in the corners, how many of the squares are white?

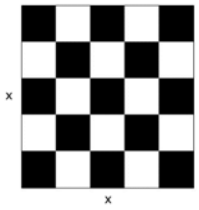

S) AR-practice task

A flower-double consists of 2 flowers above one another with tiles all around.
When you put flower-doubles in a row it looks like the figure.
For 4 flower doubles in a row, 37 slabs are needed.
If x is the number of flower-doubles then one can calculate the number of tiles y with the formula **$y = 8x + 5$**
If 4 flower-doubles are planted in a row, $y = 8 \cdot 4 + 5 = 37$ tiles are needed

How many tiles are needed if you plant 100 flower-doubles in a row?

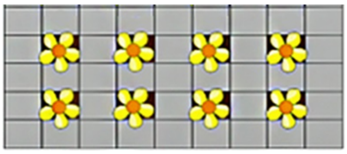

T) CMR-practice task

A flower-double consists of 2 flowers above one another with tiles all around.
When you put flower-doubles in a row it looks like the figure.
For 4 flower doubles in a row, 37 slabs are needed.

How many tiles are needed if you plant 100 flower-doubles in a row?

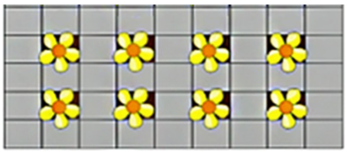

U) Transfer test task

A flower-triple consists of 3 flowers above one another with tiles all around.
When you put flower-triple in a row it looks like the figure.
For 4 flower-triples in a row, 37 slabs are needed.

How many tiles are needed if you plant 100 flower-triples in a row?

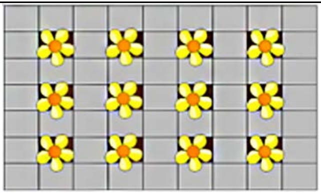

V) AR-practice task

A quilt is made of gray octagons, black squares and red and white triangles along the sides. The quilt is shaped as a square and has $x \cdot x$ eight octagons

If x is the number of octagons along one edge, the number of black squares y can be calculated by the formula **$y = x^2 - 2x + 1$**

If the quilt has $4 \cdot 4$ octagons then $y = 4^2 - 2 \cdot 4 + 1 = 9$ black squares are needed

How many black squares are needed if the quilt has 100 · 100 octagons?

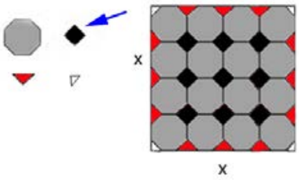

W) CMR-practice task

A quilt is made of gray octagons, black squares and red and white triangles along the sides. The quilt is shaped as a square and has $x \cdot x$ eight octagons.

If the quilt has $4 \cdot 4$ octagons then 9 black squares are needed

How many black squares are needed if the quilt has 100 · 100 octagons?

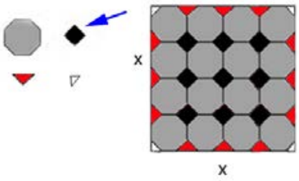

X) Transfer test task

A quilt is made of gray octagons, black squares and red and white triangles along the sides. The quilt is shaped as a rectangle and has $a \cdot b$ octagons.

If the quilt has $3 \cdot 6$ octagons, then 8 black squares are needed

How many black squares are needed if the quilt has 50 · 100 octagons?

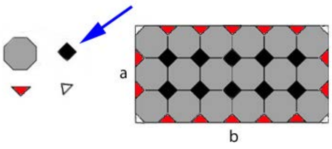

Y) AR-practice task

A street lamp illuminates the ground in a circle with a radius of 5 m.
The lamps are placed so tightly that the light cones overlap 1 m.
If x street lamps are placed in a row along a road, the illuminated road y m can be calculated by the formula **y = 9x + 1**
If you have two street lamps, a distance of y = 9 · 2 + 1 = 19 m is illuminated

How long distance will be illuminated if you put 100 lights in a row?

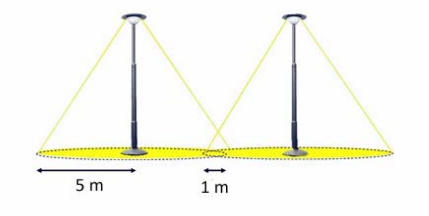

Z) CMR-practice task

A street lamp illuminates the ground in a circle with a radius 5 m.
The lamps are placed so tightly that the light cones overlap 1 m.

If you have two street lamps, a distance of 19 m is illuminated

How long distance will be illuminated if you put 100 lights in a row?

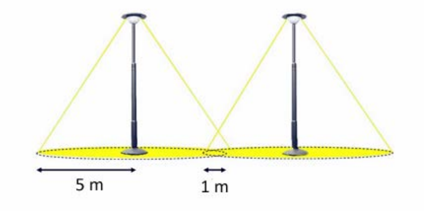

AA) Transfer test task

A street lamp illuminates the ground in a circle with a radius 6 m.
The lamps are placed so tightly that the light cones overlap 1 m.

If you have two street lamps, a distance of 23 m is illuminated

How long distance will be illuminated if you put 100 lights in a row?

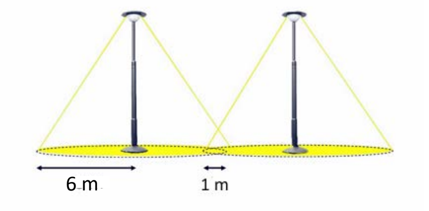

AB) AR-practice task

The following pattern is drawn. The pattern starts and ends with a square.
The sides of the square are 1 cm and the line between the squares is 2 cm.
If the color of an ink pen lasts a distance of x cm, the number of squares y that can be drawn ca be calculated by the formula **y = (x + 2) / 5**

If the ink in the pen is sufficient for 13 cm you can draw, y = (13+2)/5 = 3 squares

How many squares can you draw before the ink is sufficient for 103 cm?

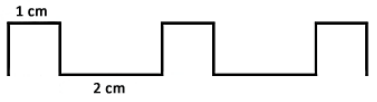

AC) CMR-practice task

The following pattern is drawn. The pattern starts and ends with a square.
The sides of the square are 1 cm and the line between the squares is 2 cm.
If the ink in the pen is sufficient for 13 cm you can draw 3 triangles
How many squares can you draw before the ink is sufficient for 103 cm??

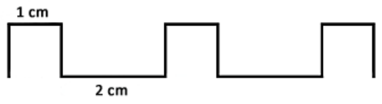

AD) Transfer test task

The following pattern is drawn. The pattern starts and ends with a triangle, open at the bottom.
The sides of the triangle are 1 cm, and the line between the triangles is 2 cm.
If the ink in the pen is sufficient for 13 cm, you can draw 3 triangles

How many triangles can you draw before the ink is sufficient for 103 cm?

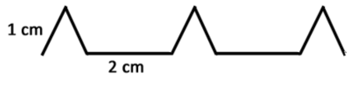

Supplementary Figure 2 (A-AD). The figure shows additional examples of practiced tests tasks and transfer test tasks
